# Neurochemical characterization of pERK-expressing spinal neurons in histamine-induced itch

**DOI:** 10.1038/srep12787

**Published:** 2015-08-07

**Authors:** Guan-Yu Jiang, Meng-Han Dai, Kun Huang, Guo-Dong Chai, Jia-Yin Chen, Ling Chen, Bing Lang, Qing-Xiu Wang, David St Clair, Colin McCaig, Yu-Qiang Ding, Ling Zhang

**Affiliations:** 1 Key Laboratory of Arrhythmias, Ministry of Education of China, East Hospital, Tongji University School of Medicine, Shanghai 200120, China; 2 Department of Anatomy and Neurobiology, Collaborative Innovation Center for Brain Science, Tongji University School of Medicine, Shanghai 200092, China; 3 School of Medical Sciences, Institute of Medical Sciences, University of Aberdeen, Foresterhill, Aberdeen AB25 2ZD, United Kingdom; 4 Department of Anesthesiology, East Hospital, Tongji University School of Medicine, Shanghai 200120, China

## Abstract

Acute itch is divided into histamine- and non-histamine-dependent subtypes, and our previous study has shown that activation of ERK signaling in the spinal dorsal horn (SDH) is required selectively for histamine-induced itch sensation. Morphological characteristics of pERK-expressing neurons are required for exploring the mechanism underlying spinal itch sensation. To investigate whether pERK-expressing neurons are supraspinally-projecting neurons, we injected Fluorogold (FG) into the ventrobasal thalamic complex (VB) and parabrachial region, the two major spinal ascending sites in rodents. A small number (1%) of pERK-positive neurons were labeled by FG, suggesting that histamine-induced activation of ERK is primarily located in local SDH neurons. We then examined the co-localization of pERK with Calbindin and Lmx1b, which are expressed by excitatory neurons, and found that more than half (58%) of pERK-positive neurons expressed Lmx1b, but no co-expression with Calbindin was observed. On the other hand, approximately 7% of pERK-positive neurons expressed GAD67, and 27% of them contained Pax2. These results support the idea that pERK-expressing neurons serve as a component of local neuronal circuits for processing itch sensation in the spinal cord.

It has been proposed that a subset of sensory neurons in the dorsal root ganglia (DRG) transmits itch-provoking stimuli from the periphery to the spinal dorsal horn (SDH), where the information is modulated via local neuronal circuits and conveyed to spinal projection neurons and transmitted to higher brain regions^1^,^2^,^3^,^4^.

Recently, progress has been made towards elucidating the molecular mechanisms underlying itch development in the DRG. The receptors of the Mas-related gene (Mrgprs) family, which are exclusively expressed in somatosensory ganglia, define a specific subpopulation of the DRG neurons mediating itch^5^,^6^,^7^. Interleukin 31 and its receptors involve the transduction of itch in the primary sensory neurons^8^. The Toll-like receptors, for example TRL7, TRL3 and TRL4, have been reported to enhance itch sensation in the DRG^9^,^10^,^11^. The neuropeptide natriuretic polypeptide b (Nppb), which is expressed in a subset of transient receptor potential vanilloid 1 (TRPV1)-expressing neurons in the DRG, specifically modulates itch behavior, as shown by the fact that the Nppb mutant mice selectively lose behavioral responses to itch- but not pain-inducing agents^12^. In addition, neurotransmitters and neuromodulators, such as substance P and glutamate, have also been reported to be involved in itch sensation in the primary sensory neurons^13^.

Meanwhile, much progress in clarifying the itch processing mechanisms in the spinal cord has been made. First, gastrin-releasing peptide receptor (GRPR)-expressing neurons in the SDH have been reported to mediate itch responses including both histamine-dependent and -independent itch^14^. Second, mice lacking BhIhb5, an atonal-related transcription factor, develop self-inflicted skin lesions and show significantly enhanced scratching responses to pruritic agents due to selective loss of a subset of inhibitory interneurons in the SDH^15^. In contrast, deletion of TR4, a testicular orphan nuclear receptor, results in a loss of excitatory interneurons in the superficial dorsal horn, and is associated with a nearly complete absence of itch behaviors and loss of certain types of nociceptive behaviors^16^. Third, it has been proposed that some spinal neurons release gastrin-releasing peptide (GRP) which acts on the GRPR-expressing neurons locally to mediate itch signal transduction^12^,^17^. In addition, we previously reported that activation of extracellular signal-related kinases (ERK) signaling in SDH neurons is selectively required for histamine-induced itch sensation^18^.

The spinal cord is the first central site for processing primary sensory information including itch, where multiple genes (factors) and neurons are implicated to constitute different functioning neuronal circuits. To uncover the highly complex neuronal circuits in the SDH, it is necessary to dissect out the neuroanatomical and neurochemical features of these spinal neurons involved in itch sensation. In the present study, we used fluorogold (FG) retrograde tracing combined with pERK immunohistochemistry to explore whether histamine-induced ERK activation is located in projection neurons, and further examined the neurochemical characteristics of pERK-positive neurons in the SDH. We found that the activation of ERK signaling in histamine-induced itch was present in a small number of supraspinally-projecting neurons, while mainly located in spinal excitatory and to a lesser extent in inhibitory interneurons.

## Result

### A small number of pERK-positive neurons projects to the thalamus

**Figure 1:**
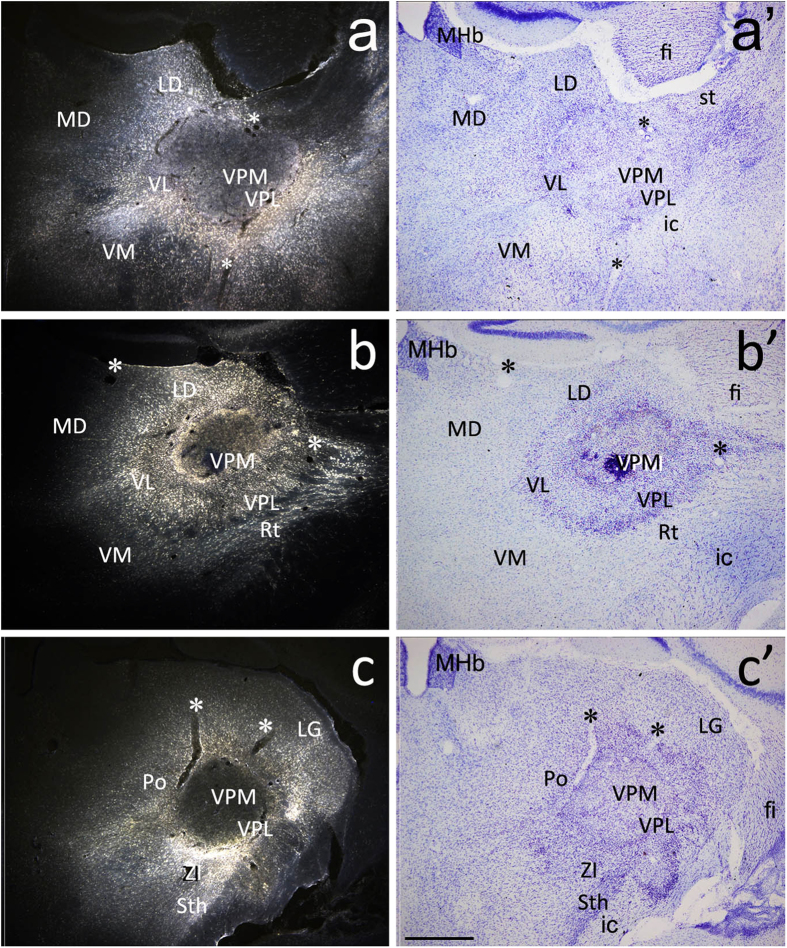
FG-injected sites in the thalamus of 3 representative mice. (**a–c**) images are obtained by ultraviolet illumination, and (**a’–c’**) are the same sites of (**a–c**), respectively. (**a’–c’**) are Nissl-stained section to show the morphology of the thalamus. Note that FG injection involves about 2/3 of the thalamus including the VB region. *indicates the same blood vessels in each pair of image. MD, mediodorsal thalamic nucleus; ic, internal capsule; fi, fimbria of the hippocampus; LD, laterodorsal thalamic nucleus; LG, lateral geniculate nucleus; MHB, medial habenular nucleus; Po, posterior thalamic nucleus; Rt, reticular thalamic nucleus; st, stria terminalis; Sth, subthalamic nucleus; VL, ventrolateral thalamic nucleus; VPM, ventral posteriomedial thalamic nucleus; VPL, ventral posteriolateral thalamic nucleus; ZI, zona inserta. Scale bar = 200 μm.

**Figure 2:**
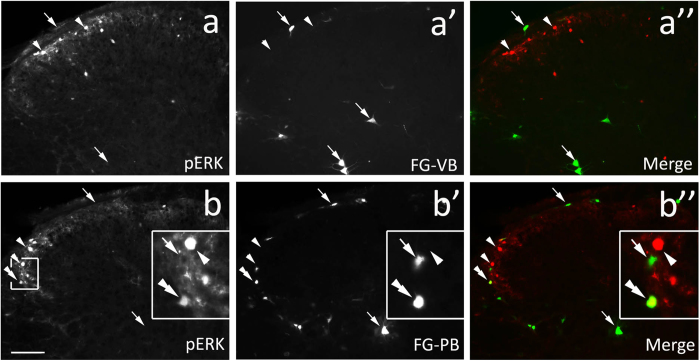
pERK immunostaining in FG-labeled spinothalamic and spinoparabrachial projection neurons in histamine-induced itch. (**a–a”**) showing pERK expression in the spinothalamic projection neurons of the SDH in histamine-induced itch. In this representative section, no neurons are labeled with both FG and pERK (see **a”**). (**b–b”**) showing pERK expression in the spinoparabrachial projection neurons of the SDH in histamine-induced itch. In this representative section, 1 FG/pERK-double labeled neuron is observed. Triangles point to pERK-positive neurons, arrows point to spinothalamic or spinoparabrachial projection neurons (**b–b”**), and the double triangles point to a FG/pERK-double labeled neuron (**b–b**”). Insets (**b–b”**) are high magnification of boxed areas. Scale bar = 100 μm.

The ventrobasal complex of the thalamus (VB) including the ventral posteriomedial nucleus and ventral posteriolateral nucleus is the major site in the spinal somatosensory ascending pathway responsible for receiving and relaying somatosensory input to the cerebral cortex^19^,^20^,^21^,^22^,^23^. To achieve the maximal labeling of spinothalamic projection neurons, a relatively larger volume of FG was injected into the thalamus centered on one side of the VB, in which the ventral posteriomedial nucleus, ventral posteriolateral nucleus, ventrolateral nucleus and posterior nucleus were involved (Fig. 1). FG-labeled spinal neurons were located mainly in laminae I, IV, and V, and were distributed more numerously in the contralateral spinal dorsal horn as reported previously^22^. Histamine was injected subcutaneously into the neck region to induce itch responses and pERK-positive neurons were primarily present in the lateral part of lamina I and outer lamina II of bilateral cervical dorsal horns^18^. We counted FG-, pERK- and FG/pERK-labeled neurons in the lateral superficial dorsal horn on the contralateral side. In a total of 133 sections from 11 mice, 209 FG-labeled and 949 pERK-positive neurons were observed (Fig. 2a–a”). Among them, only 4 neurons were labeled by both FG and pERK, showing that approximately 0.4% (4/949) of pERK-positive neurons send axons to the thalamus and approximately 1.9% (4/209) of spinothalamic projection neurons express pERK in response to histamine-induced itch.

### A small proportion of pERK-positive neurons projects to the parabrachial region

**Figure 3:**
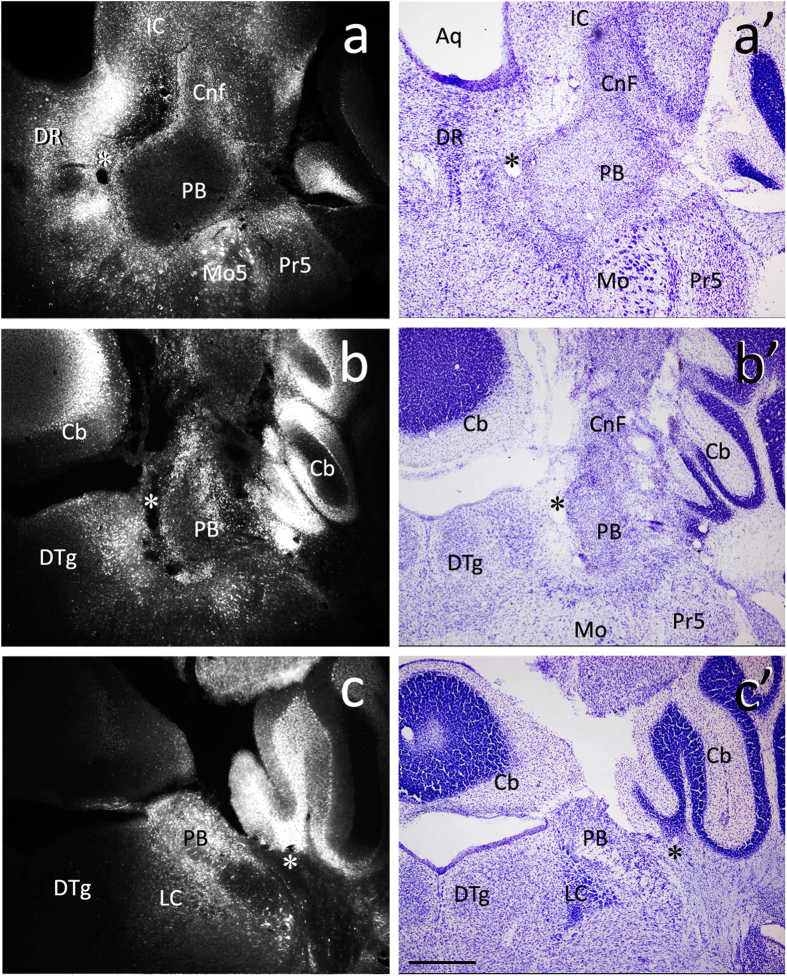
FG-injected sites in the parabrachial regions of 3 representative mice. (**a–c**) images are obtained by ultraviolet illumination, and (**a’–c’**) are the same sites of (a–c), respectively. (**a’–c’**) are Nissl-stained sections to show the morphology of the parabrachial regions. Note that FG injection involves the lateral and medial parabrachial nucleus (PB), cuneiform nucleus (Cnf) and locus coeruleus (LC). Cb, cerebellum; DTg, dorsal tegmental nucleus; Mo, motor nucleus of trigeminal nerve. Pr5, principal sensory nucleus of trigeminal nerve. *indicates the same blood vessels in each pair of image. Scale bar = 200 μm.

In addition to the thalamus, the parabrachial region particularly the lateral parabrachial nucleus is another major site for spinal ascending projection, and this pathway is also involved in the transmission of somatosensory input from the spinal cord to the thalamus and to other subcortical regions^21^,^24^,^25^,^26^,^27^,^28^. We thus set out to examine if pERK-positive neurons are spinoparabrachial projection neurons or not. FG was injected into the parabrachial region involving the lateral and medial parabrachial nuclei, cuneiform nucleus, locus coeruleus and other neighboring regions such as the periqueductal gray and cerebellum (Fig. 3). The distribution of FG-labeled neurons in the spinal cord was similar to that of FG-labeled thalamic projection reported in our previous study^28^. In a total of 156 sections from 11 mice with FG injection, 1266 pERK-positive and 549 FG-labeled neurons were observed, and 13 neurons were doubly labeled with both FG and pERK (Fig. 2b–b”), indicating that approximately 1.0% (13/1266) of pERK-positive neurons send axons to the parabrachial region, which constituted approximately 2.3% (13/549) of spinoparabrachial neurons in histamine-induced itch.

### ERK is activated mainly in local excitatory neurons

**Figure 4:**
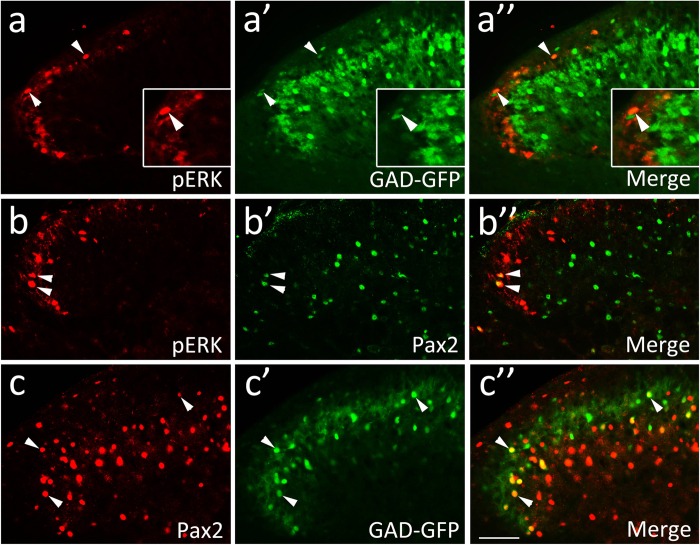
ERK activation in GAD67- and Pax2-expressing neurons in the SDH in histamine-induced itch. (**a–a”**) showing pERK expression in GFP-labeled GABAergic neurons in the lateral dorsal horn of the cervical cord in GAD67-GFP mice. Arrows point to neurons expressing both pERK and GFP. (**b–b”**) showing double immunostaining of pERK and Pax2 in the lateral dorsal horn of the cervical cord. Arrows point to GFP/pERK and Pax2/pERK-double labeled neurons. (**c–c”**) showing double immunostaining of GFP and Pax2 in the lateral dorsal horn of GAD67-GFP mice. Arrows point to GFP/Pax2-double labeled neurons. Scale bars = 100 μm.

As mentioned above, a small number of pERK-positive neurons are supraspinally-projecting neurons in histamine-induced itch. Next we investigated the neurochemical features of pERK-positive neurons in the SDH. Glutamic acid decarboxylase (GAD) 67 is an enzyme for GABA synthesis, and thus GFP can be used to reveal GABAergic interneurons reliably in GAD67-GFP knock-in mice^29^. We found that approximately 7.2% (58/795) of pERK-positive neurons were labeled with GFP (Fig. 4a–a”), showing that less than 1/10 of pERK-positive neurons are GAD67-expressing inhibitory interneurons in histamine-induced itch.

Transcription factor Pax2 is expressed in GABAergic neurons in early developing spinal cord and required for the development of spinal inhibitory neurons^30^. Thus, Pax2 may be used as a marker for inhibitory neurons, but this report also showed that Pax2 neurons in the deep SDH no longer express GAD67 and may express other inhibitory neurotransmitters such as glycine around birth. In order to know the nature of co-expression of Pax2 and GAD67 in adult SDH, we performed immunostaining of Pax2 in the GAD-GFP mice. We found that approximately 23.8% (451/1971) of Pax2-positive neurons were labeled by GFP, which corresponded to approximately 81.3% (451/562) of GAD67-expressing neurons in the superficial (laminae I-III) dorsal horn where GFP and Pax2 overlapped (Fig. 4c–c”). Furthermore, double immunostaining of pERK and Pax2 indicated that approximately 26.7% (114/434) of pERK-positive neurons were Pax2 positive, which constituted approximately 11.7% (114/952) of the total population of Pax2-positive neurons in laminae I-III (Fig. 4b–b”). If Pax2 can be used to represent a broader range of spinal inhibitory neurons as suggested by Cheng *et al.*^30^, it may be concluded that approximately 1/4 of pERK-expressing neurons are inhibitory interneurons in histamine-induced itch.

**Figure 5:**
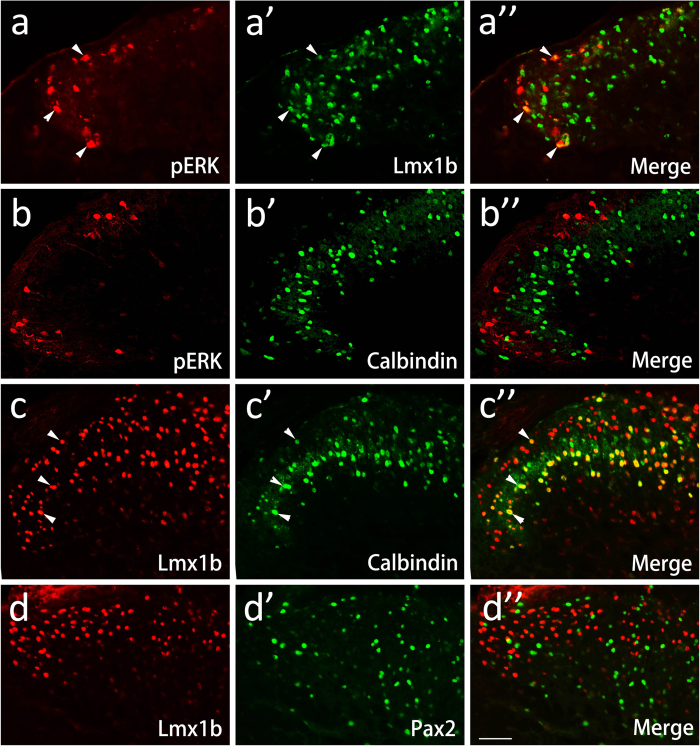
ERK activation in Lmx1b- but not in Calbindin-expressing neurons in the SDH in histamine-induced itch. (**a–a”**) showing double immunostaining of pERK and Lmx1b in the lateral dorsal horn of the cervical cord. Arrows point to neurons expressing both pERK and Lmx1b. (**b–b”**) showing double immunostaining of pERK and Calbindin in the lateral dorsal horn of the cervical cord. Note that no colocalization is observed. (**c–c”**) showing double immunostaining of Lmx1b and Calbindin in the lateral dorsal horn of the cervical cord. Note that a large number of neurons immunostained with both Lmx1b and Calbindin (arrows and yellow neurons in **c”**). (**d–d”**) showing double immunostaining of Lmx1b and Pax2 in the lateral dorsal horn of the cervical cord. Note that no colocalization of Lmx1b and Pax2 is observed. Scale bars = 100 μm.

Transcription factors Lmx1b and Tlx3 are co-expressed in spinal excitatory neurons and excluded from the population of Pax2-positive neurons in the developing spinal cord^30^,^31^. Our previous study showed that Lmx1b-positive neurons are not GABAergic in adult spinal cord^32^, further suggesting that Lmx1b can be used as a marker for spinal excitatory neurons. Double labeling of pERK and Lmx1b showed that 57.9% (277/478) of pERK-positive neurons also expressed Lmx1b (Fig. 5a–a”). However, we found no colocalization of pERK and calbindin (0/800) (Fig. 5b–b”); calbindin has been reported to be expressed by a subset of spinal excitatory interneurons^16^,^33^. Since both the Lmx1b and Calbindin are located in excitatory interneuron in the SDH, this seemingly paradoxical results led us to explore whether they label different populations of excitatory interneuron. Double immunolabeling of Lmx1b and Calbindin showed that there were 82.8% (946/1143) of Calbindin-positive neurons labeled with Lmx1b, and the double-labeled neurons were present in 56.0% (946/1689) of the total of Lmx1b-positive neurons (Fig. 5c–c”). These results demonstrate that ERK is activated only in Lmx1b- but not in Calbindin-expressing excitatory neurons in histamine-induced itch.

As mentioned above, pERK is expressed in both Lmx1b and Pax2-positive neurons, and the expressions of Lmx1b and Pax2 are mutually exclusive in the spinal neurons during embryonic development^31^. This promoted us to examine whether Lmx1b and Pax2 are also expressed in distinct neuronal populations in adult spinal cord. We co-stained Lmx1b and Pax2, and found no co-localization of these two transcription factors in the SDH (Fig. 5d–d”).

## Discussion

The SDH has different functional populations of neurons, including projection neurons and interneurons, which make up local microcircuits and integrate somatosensory inputs. According to recent findings, both supraspinally-projecting neurons and local interneurons in the spinal dorsal horn are responsible for modulation of itch at the spinal level^1^,^34^,^35^,^36^.

The projection neurons comprise neurons concentrated in lamina I and scattered throughout the deep dorsal horn. Although itch-relaying neurons have been identified in lamina I by *in vivo* physiological recording^37^,^38^, there has been no evidence showing the neurochemical characteristics of these neurons. As mentioned earlier, GRPR-expressing spinal neurons are required for itch sensation^39^ and they may be a candidate. However, GRPR-expressing neurons may not function as the projection neuron for itch transmission^40^. First, GRPR-expressing neurons are mainly distributed in lamina II but not lamina I. Second, 40–45% of substance P receptor (NK1)-expressing neurons in lamina I project to the parabrachial nucleus and about 46% of them send axons to the thalamus^22^,^28^. Namely, the majority of supraspinally- projecting neurons express NK1, but their number is not obviously changed after removal of GRPR-expressing neurons by using bombesin-saporin^39^. In addition, GRPR-positive neurons appear to be small in size, but the supraspinally-projecting neurons usually have larger cell body^40^. This reflects the possibility that GRPR-expressing neurons are interneurons in spinal neuronal circuits modulating itch information.

Our previous study showed that ERK signaling is only activated in spinal neurons in lamina I and outer lamina II, whereas many c-Fos-positive neurons are distributed throughout the SDH in histamine-induced itch^18^. This reflects the possibility that this group of neurons with ERK activation may function differently from other spinal neurons in processing itch sensation, although histamine-induced nociception cannot be fully excluded. In addition, similar ERK activation by intradermal injection of histamine is also present in anesthetized mice showing that itch stimuli, but not scratching, causes ERK phosphorylation^18^. Here we found that about 0.4% of pERK-positive neurons projected to the thalamus, the key site for transmitting input to the somatosensory cortex, and therefore may be involved in relaying itch-related information to the cortex for generation of itch perception. On the other hand, neurons from the parabrachial nucleus send projections to the insular, infralimbic and lateral frontal cortices as well as to some subcortical areas including the autonomic nuclei of the hypothalamus, amygdala and the bed nucleus of the stria terminalis^41^. On the basis of these findings, although we cannot predict reliably the functional properties of 1% of pERK-positive neurons sending axons to the parabrachial regions, it is likely that these spinoparabrachial projection neurons with ERK activation may carry itch-evoked autonomic alteration, memory, arousal and so on. However, it should be noted that this small fraction of projection neurons may not play a dominant role in transmitting itch information to higher brain regions.

It has been reported that the vast majority of neurons in the SDH have their axons remaining in the spinal cord and therefore are classified as interneurons^40^,^42^. These interneurons can be divided into two broad classes: inhibitory neurons that use GABA and/or glycine as a neurotransmitter and excitatory ones that use glutamate as their fast transmitter^42^. The interneurons make up the local complex circuits in the SDH to integrate multiple sensory modalities and considerable efforts have been made to identify interneuron populations in the SDH^40^,^42^. In addition to classifying interneurons on the basis of morphology and firing pattern, identification using arrays of specific molecular markers is used widely. Glutamic acid decarboxylase (GAD) 67 is an enzyme for synthesizing GABA and GABAergic neurons can be identified reliably with GFP in GAD67-GFP knock-in mice^29^. In line with a previous report^43^, we found that GFP-positive neurons are located mainly in laminae I-II of the SDH, and about 7% of pERK-positive neurons were GAD67-expressing neurons in histamine-induced itch. The transcription factor Pax2 serves as a marker for spinal inhibitory neurons in the early developing spinal cord^30^ and we found that it was expressed in the majority (81.3%), but not all, of GAD67-expressing neurons in the SDH, and that approximately 1/4 of pERK-positive neurons were Pax2 positive in histamine-induced itch. These results suggest that neurons with activated ERK signaling may provide inhibitory input in the local neuronal circuits for modulation of histamine-induced itch.

Whereas Pax2 labelling identifies inhibitory interneurons, Lmx1b-expressing neurons in the SDH are excitatory. Expression of Lmx1b and Pax2 are mutually exclusive in developing spinal cord^31^ and in adult SDH as shown in the present study. In addition, Lmx1b-expressing neurons are not GABAergic^32^, but co-labeled with Tlx3, an excitatory marker^30^, in the SDH^32^. Our results showed that more than half of the pERK-positive neurons expressed Lmx1b. In addition to Lmx1b, Calbindin has been used also as a marker for the excitatory interneurons in the SDH^16^,^33^. However, we did not find any pERK-positive neurons containing Calbindin although a large proportion (82.7%) of Calbindin-positive neurons also expressed Lmx1b. This indicates that ERK activation is present in a specific subset of excitatory interneurons in the SDH. Although previous studies have revealed some types of excitatory and inhibitory spinal neurons involved in itch sensation, these findings were obtained from knockout mice in which deletion of genes results in the loss of these neurons. Because of possible compensation and secondary defects caused by the gene deletion and the loss of these neurons, it is still unclear how the local neuronal circuit for the modulation of itch sensation is changed in these mice.

In conclusion, our study suggests that spinal neurons with ERK activation may play multiple roles in processing histamine-induced itch: transmitting itch-related information to higher brain regions and modulating primary itch-evoked input via local neuronal circuits in the spinal cord.

## Methods

### Animals

A total of 30 adult male ICR and 8 adult male GAD67-GFP knock-in mice^29^ were used. All experiments were performed in accordance with the guidelines of animals used in biological study of Tongji University, and were approved by the Animal Study Committee at Tongji University School of Medicine, Shanghai, China.

### FG injection

Mice were anesthetized with sodium pentobarbital (40 mg/kg, i.p.) and FG (4%, m/V; Fluorochrome, USA) was injected into the thalamus centered on the VB (approximately 0.5 μl), or parabranchial region (approximately 0.3 μl) through a fine glass micropipette with a Nanoliter 2000 pressure injection system (World Precision Instrument, USA) in 30 min. The micropipette was left *in situ* for 10 min after injection.

### Histamine-induced itch

Histamine (200 μg; Catalog number H7125, Sigma, USA) was prepared freshly in 50 μl of 0.01 M phosphate buffered saline (PBS; pH7.4) and injected intradermally into the nape to generate acute itch responses^18^. Histamine injection was performed in FG-injected mice 2 weeks after FG injection. This allows mice to recover from the operation and for FG to be transported from axon terminals to neuronal cell bodies in the spinal cord.

### Immunohistochemistry

Thirty minutes after histamine injection, mice were perfused through the ascending aorta with PBS followed by 4% paraformaldehyde in 0.1 M phosphate buffer (pH 7.4). After perfusion, spinal cords were removed and post-fixed overnight at 4 °C. Cervical cord segments (C4–C8) were excised and cut transversely into 30 μm-thick frozen sections on a cryostat. Every sixth section was collected as one set and was processed for immunofluorescence. Sections were incubated with the primary antibodies(rabbit anti-pERK1/2 antibody; 1:1000; Catalog number 9101S, Cell Signaling Technology, USA) overnight at 4 °C after antigen retrieval, and then with biotinylated donkey anti-rabbit secondary antibody (1:500; Catalog number 711065152, Jackson ImmunoResearch, USA) for 2h at room temperature, followed by incubation with Cy3-avidin (1:500; Catalog number E4142, Sigma) for 1 h at room temperature.

For double immunostaining of pERK and Lmx1b, pERK and Pax2, Lmx1b and Calbindin, Lmx1b and Pax2 (all are rabbit polyclonal antibodies), we employed the tyramide signal amplification system (TSA Plus Biotin Kit, PerkinElmer, USA)^18^,^44^,^45^. Spinal cord sections were incubated first overnight at 4 °C with rabbit anti-pERK antibody (1:100,000), a dilution at which no immunoreactivity for pERK could be detected by the conventional staining procedure. Sections then were incubated with HRP-labeled goat anti-rabbit IgG (1:100; Catalog number KC-RB-035, KangChen, China) for 3 h and with TSA Biotin Amplificatabion Reagent (1:50; PerkinElmer) for 10 min at room temperature. Signals were visualized with Cy3-conjugated streptavidin (1:500; PerkinElmer). After being washed in PBS, sections were incubated with the second primary antibody, rabbit anti-Lmx1b antibody (1:1000)^32^ or rabbit anti-Pax2 antibody (1:1000; Catalog number 716000, Invitrogen) or rabbit anti-Calbindin (1:1000; Catalog number C2724, Sigma), overnight at 4 °C, and then with Alexa Fluor 488-conjugated donkey anti-rabbit IgG (1:500; Catalog number R37118, Invitrogen) for 3 h at room temperature. Double immunostaining of Lmx1b and Calbindin or Lmx1b and Pax2 performed in the same way in which Lmx1b immunoreactivity was amplified with the TSA kit at a 1:100,000 dilution.

Given that the visualization of pERK was performed by using TSA protocol in most double labeling, some cross-reactivity may occur and skew the percentages. To exclude this, visualization of Lmx1b or Pax2 immunoreactivity was done by using TSA at a dilution of 1:100,000, and then pERK immunostaining was performed by using the conventional method at a dilution of 1:1000. Similar percentages of Lmx1b/pERK and Pax2/pERK were observed as described above.

Stained sections were observed under a Nikon fluorescence microscopy equipped with a Nikon Coolpix digital camera (DS-Ri1; Tokyo, Japan) or with laser confocal microscopy (TCS SP5 II; Leica, Germany). All images were made into figures using Adobe Photoshop (Adobe Systems Incorporated, USA) and only minor adjustments to the contrast and brightness settings were applied where necessary.

### Cell counting

Ten to fifteen sections were randomly selected from one set of sections immunostained as described above. pERK-positive neurons were located only in the lateral superficial dorsal horn due to the topographic termination pattern of primary sensory terminals from the neck region^18^. We thus counted pERK-positive, FG-labeled and pERK/FG-co-lableled neurons in the lateral half of superficial dorsal horn for evaluating the proportion of pERK-positive neurons sending axons to the supraspinal sites. For evaluating co-localization of pERK with other markers, we counted them in the whole superficial dorsal horn.

## Additional Information

**How to cite this article**: Jiang, G.-Y. *et al.* Neurochemical characterization of pERK-expressing spinal neurons in histamine-induced itch. *Sci. Rep.* **5**, 12787; doi: 10.1038/srep12787 (2015).
